# Prognosis for recipients with hepatocellular carcinoma of salvage liver transplantation versus those of primary liver transplantation: a retrospective single-center study

**DOI:** 10.1186/s40064-016-3441-5

**Published:** 2016-10-18

**Authors:** Pusen Wang, Ying Pu, Hao Li, Baojie Shi, Shengnai Zheng, Lin Zhong

**Affiliations:** 1 Department of General Surgery, Shanghai General Hospital, Shanghai Jiao Tong University School of Medicine, 100 Haining Road, Shanghai, 200080 China; 2 Department of Nursing, Shanghai General Hospital, Shanghai Jiao Tong University School of Medicine, 100 Haining Road, Shanghai, 200080 China; 3 Department of Surgery, Nanjing First Hospital, Nanjing Medical University, Nanjing, 210029 Jiangsu province China

**Keywords:** Salvage liver transplantation, Primary liver transplantation, Hepatocellular carcinoma, Prognosis

## Abstract

**Purpose:**

The prognosis for recipients with hepatocellular carcinoma (HCC) of salvage liver transplantation (SLT) versus those of primary liver transplantation (PLT) remains controversial. The objective of this study was to evaluate the clinical features and survival rate of SLT recipients.

**Methods:**

Three hundred seventy-one patients with HCC transplanted at Shanghai General Hospital, China, between October 2001 and October 2011 were separated into PLT (n = 295) and SLT (n = 76) groups. Patient characteristics and survival curves were studied by univariate and multivariate analysis. A Milan criteria-stratified survival analysis was conducted.

**Results:**

The proportions of reoperation (11.8 vs. 5.4 %, *P* = 0.047) and early postoperative mortality (11.8 vs. 4.7 %, *P* = 0.032) were higher in the SLT group than in the PLT group. Recurrence free survival (RFS) rate and overall survival (OS) rate had no statistically significant differences after stratification using Milan criteria between the PLT group and SLT group. Alphafetoprotein >400 ng/mL (*P* = 0.011), microscopic vascular invasion (MVI) (*P* < 0.001), tumor node metastasis (TNM) staging (*P* = 0.006), and out of Milan criteria (*P* < 0.001) were independent risk factors for RFS, while MVI (*P* < 0.001), TNM staging (*P* = 0.009), and out of Milan criteria (*P* = 0.003) were factors for OS. In the multivariate logistic regression analysis, HCC recurrence was associated with MVI (OR = 4.196 [2.538–6.936], *P* < 0.001), and out of Milan criteria (OR = 2.704 [1.643–4.451], *P* < 0.001).

**Conclusions:**

Our retrospective, single-center study demonstrated that SLT increases surgical difficulty; however, it has good post-transplantation OS and is a feasible alternative after HCC recurrence within Milan criteria.

## Background

Hepatocellular carcinoma (HCC) is the seventh most common cancer and the third leading cause of cancer-related death worldwide (Yang and Roberts 2010), with an estimated 782,000 liver cancer cases and 746,000 liver cancer-related deaths in 2012 (Siegel et al. 2014). In general, HCC incidence and mortality have been slowly decreasing in previous areas of relatively high incidence, including China and Japan; most HCC cases (>80 %) now occur in sub-Saharan Africa and in Eastern Asia, with typical incidence rates of >20 per 100,000 individuals (El-Serag 2012). Despite various therapeutic options, such as liver resection (LR), radiofrequency ablation, and transcatheter hepatic arterial chemoembolization, the prognosis remains generally poor, leading to 500,000 deaths per year (Maluccio and Covey 2012).

Liver transplantation (LT) is advisable in patients with HCC and decompensated cirrhosis with excellent results in terms of overall and recurrence-free survival (OS and RFS, respectively) in selected patients (Mazzaferro et al. 1996; Befeler et al. 2005; Lee et al. 2010); however, organ shortage is a worldwide problem, especially in China, because of the large population base, people’s financial situation, and legal limitations. As a result, the risk of drop-out for tumor progression and the deterioration of the patients’ clinical conditions are an ongoing problem (Yao et al. 2002, 2004; Guerrini et al. 2009; Freeman et al. 2006; Li and Neuberger 2009).

Salvage liver transplantation (SLT) is a protocol that offers LR first and subsequent liver transplantation for tumor recurrence or deteriorating liver function (Majno et al. 2000). For patients with small, solitary HCC and with preserved hepatic function, it has been reported that SLT has a long-term survival rate similar to that of those who directly undergo primary liver transplantation (PLT) (Majno et al. 2000; Cherqui et al. 2009; Wu et al. 2012; Hu et al. 2012). SLT, which reduces the risk of HCC progressing during the time awaiting transplantation, might offer a good strategy for relieving patients with a good prognosis and also alleviate the burden on the donor organ pool (Wu et al. 2012; Fan et al. 2011).

Our institution (Shanghai General Hospital) adopted and conducted SLT strategy since it was first proposed by Majno et al. (2000). To aid in the assessment of the feasibility of SLT and its indications, we retrospectively reviewed and compared the clinical features and survival rates of patients undergoing SLT with those undergoing PLT in our institution between 2001 and 2011.

## Results

### Clinical demographics and follow up data

The main demographics of the 371 patients are listed in Table 1. The PLT group had a significantly higher percentage of alphafetoprotein (AFP) >400 ng/mL (*P* = 0.008) than the SLT group. Moreover, the percentage of patients with Child–Pugh B, C was higher in the PLT group than in the SLT group (*P* = 0.002). Tumor characteristics and operating parameters between the two groups are listed in Table 2. The percentage of patients with the largest tumor >3.0 cm in diameter (*P* < 0.001), microscopic vascular invasion (MVI) (*P* = 0.001), or recurrence (*P* < 0.001) were higher in the PLT group, while that of multinodular tumors was lower (*P* = 0.018). Moreover, there were more patients with noncompliance with Milan criteria in the PLT group, but the difference was not significant (*P* > 0.05). Among operating parameters, the proportion of reoperation (11.8 vs. 5.4 %, respectively; *P* = 0.047) and early postoperative mortality (11.8 vs. 4.7 %, respectively; *P* = 0.032) were higher in SLT group than in the PLT group, which indicated that SLT might increase surgical difficulty and risk. The mean and median follow-up time was 32.43 months and 19.53 months, respectively (range 0.03–146.60 months). The 1-, 3- and 5-year survival rates were 69.2, 51.8 and 46.6 %, respectively. One hundred seventy-eight (48.0 %) patients died during follow-up. The most common cause of death in these patients was HCC recurrence.

**Table 1:** Demographics of the patients

	PLT group (n = 295)	SLT group (n = 76)	*P* value
Age, years	48.3 ± 8.5	48.5 ± 8.6	0.870
Gender (male/female)	263/32	68/8	0.936
Blood type (A/B/AB/O)	95/82/38/80	32/16/10/18	0.385
Preoperative MELD score	11.0 ± 4.4	10.6 ± 5.8	0.570
AFP >400 ng/mL, no (%)	101 (34.2)	14 (18.4)	0.008
Underlying liver disease, no (%)			
HBV	277 (93.9)	68 (89.5)	
HCV	2 (0.7)	2 (2.6)	
Autoimmune hepatitis	11 (3.7)	4(5.3)	
Wilson’s disease	1 (0.3)	0 (0)	
Chronic alcoholism	2 (0.7)	0 (0)	
Cryptogenic	2 (0.7)	2 (2.6)	0.272
Child–Pugh grading (A/B/C)	150/109/36	56/14/6	0.002

*PLT* primary liver transplantation, *SLT* salvage liver transplantation, *MELD* model for end-stage liver disease, *AFP* alpha-fetoprotein, *HBV* hepatitis B virus, HCV, hepatitis C virus, *HCC* hepatocellular carcinoma

**Table 2:** Tumor characteristics and operative parameters

	PLT group (n = 295)	SLT group (n = 76)	*P* value
Diameter of largest tumor >3 cm, no (%)	189 (64.1)	30 (39.5)	<0.001
Multinodular, no (%)	126 (42.7)	44 (57.9)	0.018
TNM staging, (1–2/3–4)	155/140	36/40	0.442
Out of Milan criteria, no (%)	194 (65.8)	46 (60.5)	0.394
Poorly differentiated tumor, no (%)	56 (19.0)	15 (19.7)	0.882
MVI, no (%)	155 (52.5)	24 (31.6)	0.001
Operative time, hour	6.0 ± 2.0	7.0 ± 2.5	0.260
Blood loss, mL	2010 ± 1500	2450 ± 2375	0.052
Packed red cell transfusion, U	8.0 ± 10.0	8.5 ± 8.8	0.078
Reoperation, no (%)	16 (5.4)	9 (11.8)	0.047
Early postoperative mortality, no (%)	14 (4.7)	9 (11.8)	0.032
Recurrence, no (%)	124 (42.0)	10 (13.2)	<0.001

*PLT* primary liver transplantation, *SLT* salvage liver transplantation, *TNM* tumor node metastasis, *MVI* microscopic vascular invasion

### Patient survival: Kaplan–Meier survival curves and Cox hazard regression analysis

**Fig. 1:**
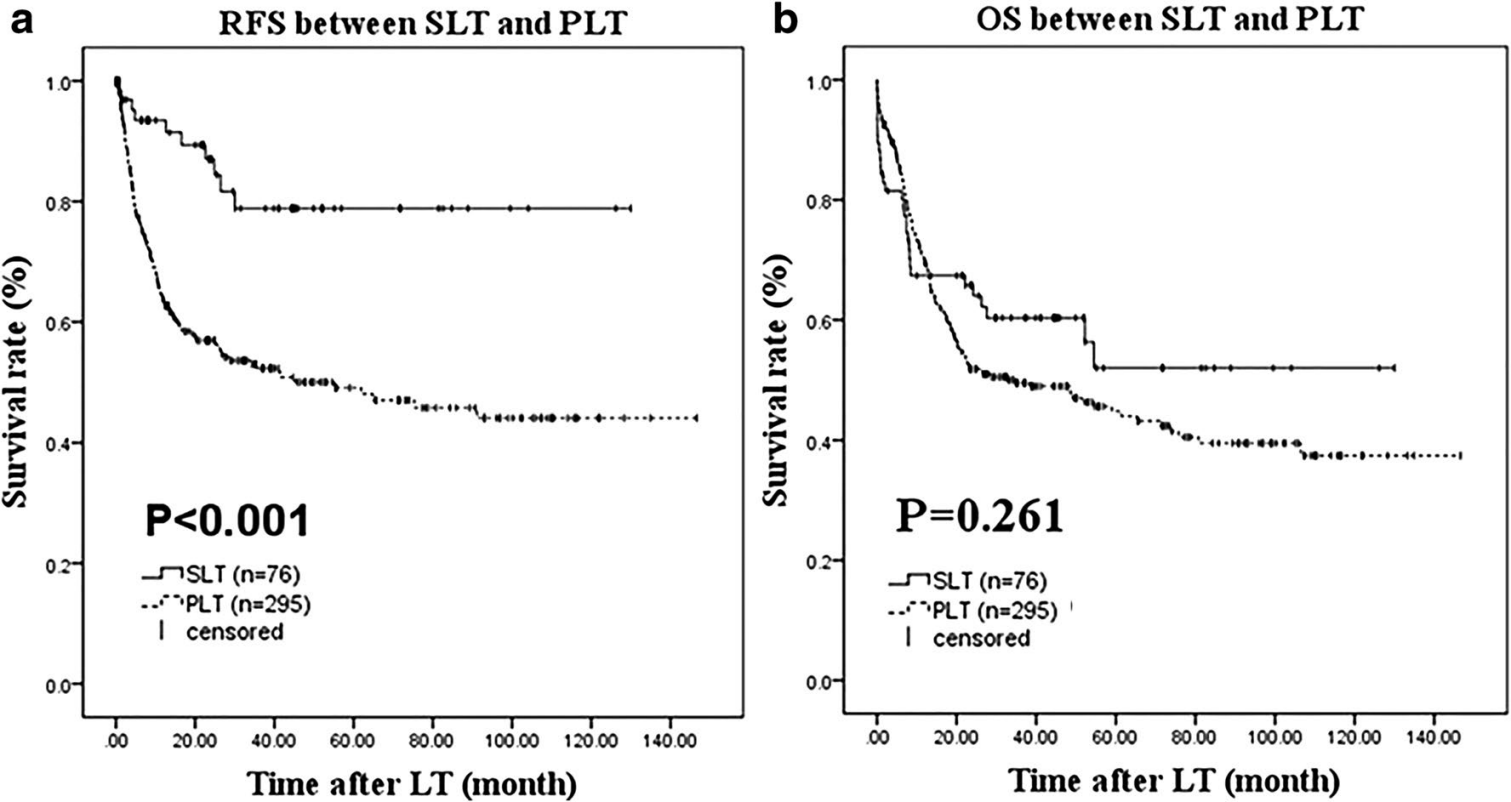
Kaplan–Meier survival estimates of RFS (**a**) and OS (**b**) between SLT and OLT

Recurrence-free survival and OS between the SLT and PLT groups were analyzed with Kaplan–Meier survival estimates as well as the log-rank test. Interestingly, the RFS rate, but not the OS rate, was significantly lower in the PLT group (Fig. 1a, *P* < 0.001 and Fig. 1b, *P* > 0.05, respectively). Univariate and multivariate models were used to identify factors associated with RFS and OS for all LT patients. All variables with *P* < 0.05 in the univariate analysis (data not shown) were placed into the multivariate Cox regression model. As shown in Table 3, AFP >400 ng/mL (*P* = 0.011), MVI (*P* < 0.001), tumor-node-metastasis (TNM) staging (*P* = 0.006), and noncompliance with Milan criteria (*P* < 0.001) were independent risk factors for RFS, while MVI (*P* < 0.001), TNM staging (*P* = 0.009), and noncompliance with Milan criteria (*P* = 0.003) were risk factors for OS.

**Table 3:** Multivariate Cox regression analysis of all LT patients

	HR	95 % CI for HR		*P* value
		Lower	Upper	
Recurrence-free survival				
AFP >400 ng/mL	1.581	1.109	2.255	0.011
MVI	5.558	3.540	8.724	<0.001
TNM staging	2.513	1.298	4.862	0.006
Out of Milan criteria	2.945	1.946	4.457	<0.001
Overall survival				
MVI	3.798	2.659	5.425	<0.001
TNM staging	1.499	1.104	2.034	0.009
Out of Milan criteria	1.673	1.197	2.338	0.003

*CI* confidence interval, *HR* hazard ratio, *AFP* alpha-fetoprotein, *MVI* microscopic vascular invasion, *TNM* tumor node metastasis

**Fig. 2:**
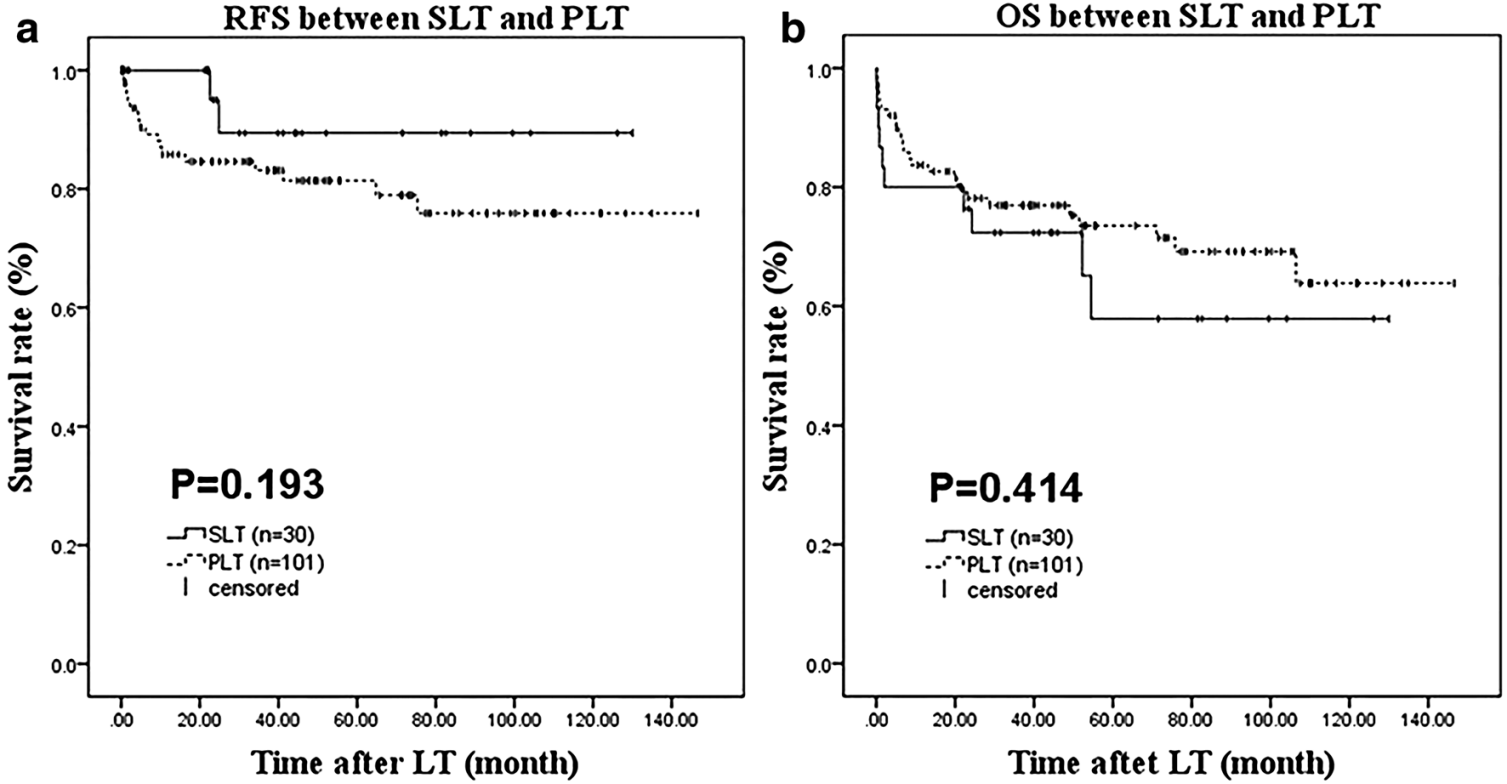
Kaplan–Meier survival estimates of RFS (**a**) and OS (**b**) between SLT and OLT within Milan criteria

In consideration of the results of multivariate analysis, and more patients with the largest tumor being >3.0 cm in diameter, MVI, and noncompliance with Milan criteria in the PLT group, a Milan criteria-stratified survival analysis was conducted. Among the 371 patients, 131 (35.3 %) met the Milan criteria—101 (34.2 %) in the PLT group and 30 (39.5 %) in the SLT group. As shown in Fig. 2, there were no statistically significant differences in either RFS or OS between the SLT and PLT groups (*P* = 0.193 and *P* = 0.414, respectively; Fig. 2a, b).

### Risk factors of recurrence: multivariate logistic regression analysis

Univariate and multivariate models were used to calculate the factors associated with HCC recurrence for all LT patients. All variables with *P* < 0.05 in the univariate analysis (data not shown) were put into the multivariate logistic regression model. In the multivariate logistic regression analysis, HCC recurrence was significantly associated with the following factors: MVI (odds ratio [OR] = 4.196 [2.538–6.936], *P* < 0.001) and noncompliance with Milan criteria (OR = 2.704 [1.643–4.451], *P* < 0.001) (Table 4). SLT or PLT itself was not an independent risk factor of HCC recurrence.

**Table 4:** Multivariate logistic regression analysis of risk factors for HCC recurrence

	OR	95 % CI for OR		*P* value
		Lower	Upper	
MVI	4.196	2.538	6.936	<0.001
Out of Milan criteria	2.704	1.643	4.451	<0.001

*CI* confidence interval, *OR* odds ratio, *HCC* hepatocellular carcinoma, *MVI* microscopic vascular invasion

## Discussion

Whether SLT and PLT recipients have a similar prognosis remains controversial. In our single-center retrospective study, we demonstrated that OS and RFS of SLT recipients was not different from those of PLT recipients within Milan criteria, even though SLT increases surgical difficulties and risks.

Hepatocellular carcinoma is one of the most common cancers in China, with a relatively high mortality (El-Serag 2012). Theoretically, LT is the most effective option for HCC because it not only radically removes the tumor but also cures the underlying hepatic disease (Befeler et al. 2005); however, the shortage of organs greatly limits the application of LT to all patients. SLT, raised by Majno et al. (2000), might relieve disease progression in patients waiting for LT and could reduce the number of transplantations required (Chan et al. 2014).

The outcome for recipients of SLT has been studied for many years since it was first proposed in 2000. Some studies have indicated that SLT has a prognosis similar to that of PLT (Majno et al. 2000; Cherqui et al. 2009; Wu et al. 2012; Hu et al. 2012), while others have yielded contradictory findings (Adam et al. 2003; Guerrini et al. 2014). In the study conducted by Adam et al. (2003), the poor prognosis after SLT compared to that of PLT might be related to the relatively higher surgical mortality and intraoperative bleeding because of more technical difficulties in secondary LT. Moreover, only 17 patients with HCC recurrence were included in the SLT group. Another recent study conducted by Guerrini (2014) demonstrated that SLT achieved good post-transplantation survival, but the outcome of SLT remains inferior to PLT in the intention-to-treat analysis. RFS and OS between SLT and PLT in their study showed no significant differences, which is consistent with our results.

An interesting observation from this study is that the RFS rate was significantly lower in the PLT group (Fig. 1a). The explanation for this might be that the PLT group in our study appeared to have had more aggressive HCC, as indicated by the greater proportion of tumors >3.0 cm in diameter, MVI, and recurrence. Accordingly, a Milan criteria-stratified survival analysis was conducted and the results showed no significant differences between SLT and PLT, which indicated that SLT for HCC recurrence after liver resection is a feasible alternative and does not significantly impair survival compared to that of PLT. Some studies have demonstrated that SLT increases surgical difficulty (Wu et al. 2012; Hu et al. 2012). In our study, the SLT group did have a remarkably higher proportion of subsequent surgery and early postoperative mortality, and with longer operative time, more blood loss, and packed red cell transfusions, although the differences were not significant. Peritoneal adhesions caused by previous upper abdominal surgery were the main reason for increased surgical difficulty. The adhesions usually occur near the intestine and the omentum, according to our experience and previous study, and can be reduced by laparoscopic surgery (Wu et al. 2012). We believe that the reoperation rate and early postoperative mortality can be reduced by an accumulation of clinician experience as the number of SLT cases increases. In the Cox hazard regression analysis and multivariate logistic regression analysis, MVI was an independent risk factor for RFS, OS, and recurrence, but SLT or PLT was not. However, the MVI data at primary LR was not analyzed in our study. Similarly, Roayaie et al. (2009) reported that MVI could accurately predict the risk of recurrence and survival of patients after resection of HCC. Accordingly, the valuable MVI data might possibly predict tumor recurrence to some extent.

There were some main limitations to our study. First, there was a lack of intention-to-treat analysis. We did not have the adequate LR data on some patients because the hepatectomy was not conducted in our institution. Second, we conducted a single-center, retrospective study, which inevitably shared the limitations with the analyses of observational data. The extended period over which patients were selected for inclusion in the study was an additional limitation. With advances in surgical techniques, improved management after surgery, and more reasonable immunosuppressive therapy, the clinical prognosis has changed since the period of selection of study subjects, resulting in heterogeneity among these patients.

## Conclusion

In this study, we demonstrated that SLT has good OS post-transplantation and is a feasible alternative for HCC recurrence within Milan criteria. Organ shortages and tumor progression considered, SLT is an alternative strategy for the treatment of patients with HCC.

## Methods

### Ethics approval

For this retrospective study formal consent is not required. This study was approved by the Institutional Review Board Liver Transplantation Surgery, Shanghai General Hospital, Shanghai, China under the guidelines of the Ethics Committee of the hospital, and the Declaration of Helsinki (World Medical Association declaration of Helsinki. Recommendations guiding physicians in biomedical research involving human subjects 1997).

### Patients

A retrospective review of the prospectively maintained database at Shanghai General Hospital, Shanghai Jiao Tong University School of Medicine, China, was conducted. Three hundred seventy-one (331 males) HCC patients treated with LT between 2001 and 2011 were enrolled. The mean age of patients in our sample was 48 years (range 16–65 years). All HCC diagnoses were based on histology results. The mean follow-up time was 32.43 months (range 0.03–146.60 months). The follow-up included alpha-fetoprotein and an ultrasound one to four times each month, and a thoracic-abdominal computer tomography scan every 3.0–6.0 months during the first 2.0 years and annually thereafter. Patients with HCC fulfilling Hangzhou criteria (Xu et al. 2016) (tumour burden ≤8 cm regardless of AFP and differentiation, or tumour burden >8 cm but AFP ≤400 ng/mL and well-moderate differentiation) who received LT were included in the study. Although this study included patients using Hangzhou criteria rather than Milan criteria as previous studies (Fuks et al. 2012; Sala et al. 2004; Ferrer-Fabrega et al. 2016), stratification analysis using Milan criteria was conducted. The exclusion criteria were (1) had undergone LT more than once; (2) had living donors or split-liver donors; (3) diagnosed with other malignancies in addition to HCC (cholangiocarcinoma, carcinoma of gallbladder, or mixed carcinoma); (4) had transplantation without recurrence after liver resection for HCC; (5) had tumor thrombosis of the main trunk of blood vessel (portal vein and hepatic vein); or (6) had missing pathology data.

### Data collection

Patient baseline and clinical data comprising age, sex, blood type, pre-LT model for end-stage liver disease (MELD) score (Malinchoc et al. 2000), pre-LT serum AFP level (stratification according to previous research) (Zheng et al. 2008), underlying liver disease, Child–Pugh status (Pugh et al. 1973), diameter of largest tumor, multinodular tumors, TNM staging (according to International Union Against Cancer/American Joint Committee on Cancer criteria), noncompliant with Milan criteria (Mazzaferro et al. 2008), histologic grade (differentiated [well differentiated + moderately differentiated] and poorly differentiated), MVI at LT, operative time, blood loss, packed red cell transfusion, reoperation, early postoperative mortality (defined as death within the first 90 days post-surgery), HCC recurrence, OS, and RFS were recorded. OS was defined as time from date of LT to death due to any cause. RFS was defined as the time from LT to the date of confirmed recurrence (Raza et al. 2015).

### Statistical analyses

All statistical analyses were performed using SPSS ver.19.0 statistical software (SPSS Inc., Chicago, IL, USA). Continuous data were expressed as the mean ± standard deviation (SD) or median (range), and discrete variables as frequencies. Categorical variables were compared using the Pearson’s *χ* ^*2*^ test or Fisher’s exact test, whereas continuous variables were calculated with Student’s *t* test or Mann–Whitney test. Survival rates were assessed using Kaplan–Meier analysis and compared using the log-rank test. Recipient and tumor variables were calculated with univariate analysis and multivariate analysis for OS and RFS, respectively, using the Cox proportional hazard regression model. The logistic regression model was applied to investigate predictors of HCC recurrence after transplantation. The final models were determined by placing all variables with *P* < 0.05 from the univariate analysis into the multivariate Cox regression analysis and by using a forward stepwise method. Statistical significance was established as *P* < 0.05.

## Abbreviations

HCC: hepatocellular carcinoma
LR: liver resection
SLT: salvage liver transplantation
PLT: primary liver transplantation
RFS: recurrence free survival
OS: overall survival
MVI: microscopic vascular invasion
TNM: tumor node metastasis
AFP: alphafetoprotein
MELD: model for end-stage liver disease
HBV: hepatitis B virus
HCV: hepatitis C virus
CI: confidence interval
HR: hazard ratio
OR: odds ratio

## References

[CR1] Adam R, Azoulay D, Castaing D, Eshkenazy R, Pascal G, Hashizume K, Samuel D, Bismuth H (2003). Liver resection as a bridge to transplantation for hepatocellular carcinoma on cirrhosis: a reasonable strategy?. Ann Surg.

[CR2] Sala M, Fuster J, Llovet JM, Navasa M, Sole M, Varela M, Pons F, Rimola A, Garcia-Valdecasas JC, Bru C, Bruix J, Barcelona Clinic Liver Cancer G (2004). High pathological risk of recurrence after surgical resection for hepatocellular carcinoma: an indication for salvage liver transplantation. Liver Transpl.

[CR3] Befeler AS, Hayashi PH, Di Bisceglie AM (2005). Liver transplantation for hepatocellular carcinoma. Gastroenterology.

[CR4] Chan DL, Alzahrani NA, Morris DL, Chua TC (2014). Systematic review of efficacy and outcomes of salvage liver transplantation after primary hepatic resection for hepatocellular carcinoma. J Gastroenterol Hepatol.

[CR5] Cherqui D, Laurent A, Mocellin N, Tayar C, Luciani A, Van Nhieu JT, Decaens T, Hurtova M, Memeo R, Mallat A, Duvoux C (2009). Liver resection for transplantable hepatocellular carcinoma: long-term survival and role of secondary liver transplantation. Ann Surg.

[CR6] El-Serag HB (2012) Epidemiology of viral hepatitis and hepatocellular carcinoma. Gastroenterology 142 (6):1264–1273 e1261. doi: 10.1053/j.gastro.2011.12.061 10.1053/j.gastro.2011.12.061 PMC3338949 22537432

[CR7] Fan ST, Poon RT, Yeung C, Lam CM, Lo CM, Yuen WK, Ng KK, Liu CL, Chan SC (2011). Outcome after partial hepatectomy for hepatocellular cancer within the Milan criteria. Br J Surg.

[CR8] Ferrer-Fabrega J, Forner A, Liccioni A, Miquel R, Molina V, Navasa M, Fondevila C, Garcia-Valdecasas JC, Bruix J, Fuster J (2016). Prospective validation of ab initio liver transplantation in hepatocellular carcinoma upon detection of risk factors for recurrence after resection. Hepatology.

[CR9] Freeman RB, Edwards EB, Harper AM (2006). Waiting list removal rates among patients with chronic and malignant liver diseases. Am J Transplant.

[CR10] Fuks D, Dokmak S, Paradis V, Diouf M, Durand F, Belghiti J (2012). Benefit of initial resection of hepatocellular carcinoma followed by transplantation in case of recurrence: an intention-to-treat analysis. Hepatology.

[CR11] Guerrini GP, Pleguezuelo M, Maimone S, Calvaruso V, Xirouchakis E, Patch D, Rolando N, Davidson B, Rolles K, Burroughs A (2009). Impact of tips preliver transplantation for the outcome posttransplantation. Am J Transplant.

[CR12] Guerrini GP, Gerunda GE, Montalti R, Ballarin R, Cautero N, De Ruvo N, Spaggiari M, Di Benedetto F (2014). Results of salvage liver transplantation. Liver Int.

[CR13] Hu Z, Zhou J, Xu X, Li Z, Zhou L, Wu J, Zhang M, Zheng S (2012). Salvage liver transplantation is a reasonable option for selected patients who have recurrent hepatocellular carcinoma after liver resection. PLoS ONE.

[CR14] Lee KK, Kim DG, Moon IS, Lee MD, Park JH (2010). Liver transplantation versus liver resection for the treatment of hepatocellular carcinoma. J Surg Oncol.

[CR15] Li KK, Neuberger J (2009). The management of patients awaiting liver transplantation. Nat Rev Gastroenterol Hepatol.

[CR16] Majno PE, Sarasin FP, Mentha G, Hadengue A (2000). Primary liver resection and salvage transplantation or primary liver transplantation in patients with single, small hepatocellular carcinoma and preserved liver function: an outcome-oriented decision analysis. Hepatology.

[CR17] Malinchoc M, Kamath PS, Gordon FD, Peine CJ, Rank J, ter Borg PC (2000). A model to predict poor survival in patients undergoing transjugular intrahepatic portosystemic shunts. Hepatology.

[CR18] Maluccio M, Covey A (2012). Recent progress in understanding, diagnosing, and treating hepatocellular carcinoma. CA.

[CR19] Mazzaferro V, Regalia E, Doci R, Andreola S, Pulvirenti A, Bozzetti F, Montalto F, Ammatuna M, Morabito A, Gennari L (1996). Liver transplantation for the treatment of small hepatocellular carcinomas in patients with cirrhosis. N Engl J Med.

[CR20] Mazzaferro V, Chun YS, Poon RT, Schwartz ME, Yao FY, Marsh JW, Bhoori S, Lee SG (2008). Liver transplantation for hepatocellular carcinoma. Ann Surg Oncol.

[CR21] Pugh RN, Murray-Lyon IM, Dawson JL, Pietroni MC, Williams R (1973). Transection of the oesophagus for bleeding oesophageal varices. Br J Surg.

[CR22] Raza SJ, Wilson T, Peabody JO, Wiklund P, Scherr DS, Al-Daghmin A, Dibaj S, Khan MS, Dasgupta P, Mottrie A, Menon M, Yuh B, Richstone L, Saar M, Stoeckle M, Hosseini A, Kaouk J, Mohler JL, Rha KH, Wilding G, Guru KA (2015). Long-term oncologic outcomes following robot-assisted radical cystectomy: results from the International Robotic Cystectomy Consortium. Eur Urol.

[CR23] Roayaie S, Blume IN, Thung SN, Guido M, Fiel MI, Hiotis S, Labow DM, Llovet JM, Schwartz ME (2009). A system of classifying microvascular invasion to predict outcome after resection in patients with hepatocellular carcinoma. Gastroenterology.

[CR24] Siegel R, Ma J, Zou Z, Jemal A (2014). Cancer statistics, 2014. CA.

[CR25] World Medical Association declaration of Helsinki (1997). Recommendations guiding physicians in biomedical research involving human subjects. Jama.

[CR26] Wu L, Hu A, Tam N, Zhang J, Lin M, Guo Z, He X (2012). Salvage liver transplantation for patients with recurrent hepatocellular carcinoma after curative resection. PLoS ONE.

[CR27] Xu X, Lu D, Ling Q, Wei X, Wu J, Zhou L, Yan S, Wu L, Geng L, Ke Q, Gao F, Tu Z, Wang W, Zhang M, Shen Y, Xie H, Jiang W, Wang H, Zheng S (2016). Liver transplantation for hepatocellular carcinoma beyond the Milan criteria. Gut.

[CR28] Yang JD, Roberts LR (2010) Epidemiology and management of hepatocellular carcinoma. Infect Dis Clin North Am 24 (4):899–919, viii. doi: 10.1016/j.idc.2010.07.004 10.1016/j.idc.2010.07.004 PMC3949429 20937457

[CR29] Yao FY, Bass NM, Nikolai B, Davern TJ, Kerlan R, Wu V, Ascher NL, Roberts JP (2002). Liver transplantation for hepatocellular carcinoma: analysis of survival according to the intention-to-treat principle and dropout from the waiting list. Liver Transpl.

[CR30] Yao FY, Bass NM, Ascher NL, Roberts JP (2004). Liver transplantation for hepatocellular carcinoma: lessons from the first year under the Model of End-Stage Liver Disease (MELD) organ allocation policy. Liver Transpl.

[CR31] Zheng SS, Xu X, Wu J, Chen J, Wang WL, Zhang M, Liang TB, Wu LM (2008). Liver transplantation for hepatocellular carcinoma: hangzhou experiences. TransplantaFtion.

